# Crystal structure of 1-ferrocenyl-2-(4-methyl­benzo­yl)spiro­[11*H*-pyrrolidizine-3,11′-indeno[1,2-*b*]quinoxaline]

**DOI:** 10.1107/S1600536814017644

**Published:** 2014-08-09

**Authors:** Kuppan Chandralekha, Deivasigamani Gavaskar, Adukamparai Rajukrishnan Sureshbabu, Srinivasakannan Lakshmi

**Affiliations:** a Research Department of Physics, S. D. N. B. Vaishnav College for Women, Chromepet, Chennai 600 044, India; b Department of Organic Chemistry, University of Madras, Guindy Campus, Chennai 600 025, India

**Keywords:** crystal structure, ferrocen­yl, pyrrolidizine, quinoxaline, hydrogen bonds

## Abstract

In the title compound, [Fe(C_5_H_5_)(C_34_H_28_N_3_O)], the four-fused-rings system of the 11*H*-indeno­[1,2-*b*]quinoxaline unit is approximately planar [maximum deviation = 0.167 (4) Å] and forms a dihedral angle of 37.25 (6)° with the plane of the benzene ring of the methyl­benzoyl group. Both pyrrolidine rings adopt a twist conformation. An intra­molecular C—H⋯O hydrogen bond is observed. In the crystal, mol­ecules are linked by C—H⋯O hydrogen bonds and weak C—H⋯π inter­actions, forming double chains extending parallel to the *c* axis.

## Abstract 2

In the title compound the four-fused-rings system is approximately planar and the pyrrolidine rings of the pyrrolidizine fragment adopt a twist conformation. In the crystal, mol­ecules are linked by C—H⋯O hydrogen bonds and C—H⋯π inter­actions, forming double-chains parallel to the *c* axis.

## Chemical Context   

Spiro­oxindoles are an important class of naturally occurring substances characterized by highly pronounced biological properties (Sureshbabu & Raghunathan, 2008 ▶). Ferrocene derivatives have anti­malarial (Biot *et al.*, 2004 ▶) and anti­bacterial (Chohan, 2002 ▶) activities. The use of ferrocene in bio-organometallic chemistry has promising applications since ferrocene is a stable non-toxic compound and has good redox properties (Fouda *et al.*, 2007 ▶). Ferrocenyloxindoles have also been found to have anti­cancer (Silva *et al.*, 2010 ▶) and anti­proliferative activities (Gasser *et al.*, 2011 ▶).
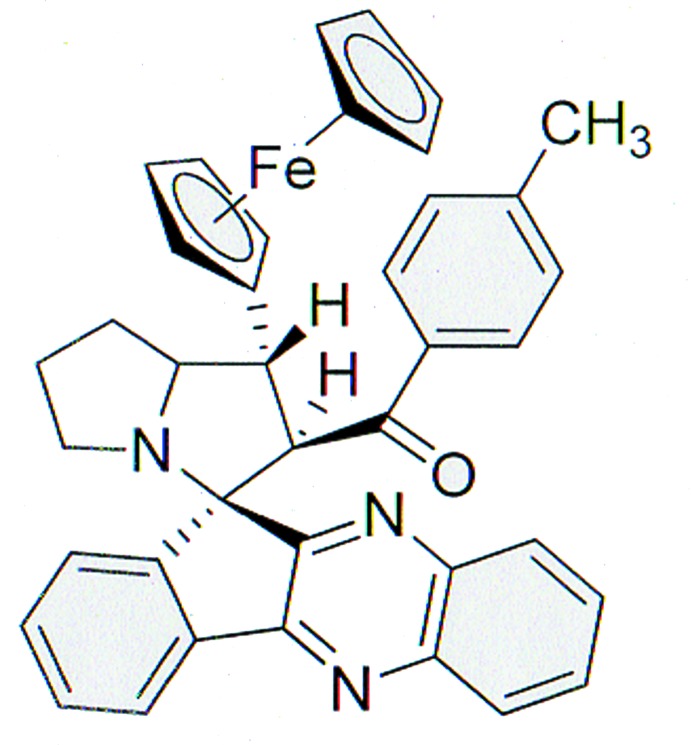


The synthesis of novel ferrocenyl-spiro-indanedione-*N*-methyl­pyrrolidines by employing various unusual ferrocene derivatives as efficient 2π-components in 1,3-dipolar cyclo­addition reactions of azomethine ylides demonstrate that ferrocene-derived dipo­lar­o­philes can further be exploited for the synthesis of a variety of complex heterocycles through cyclo­addition reactions (Sureshbabu *et al.*, 2009 ▶). A wide range of substituted pyrrolizidine scaffolds offers a high level of functional, structural and stereochemical diversity. It has been demonstrated that multicomponent reactions (MCR) could be used for the synthesis of novel ferrocene-grafted di­spiro­pyrrolidine and pyrrolizidine scaffolds through one-pot three-component inter­molecular [3 + 2] cyclo­addition of azomethine ylides with an unusual ferrocene Baylis–Hillman adduct (Kathiravan & Raghunathan, 2009 ▶). The one-pot four-component cyclo­addition reaction method was used to synthesize substituted pyrrolizidines containing ferrocene and a spiro-indeno­quinoxaline moiety of biological significance (Sureshbabu *et al.*, 2012 ▶). In view of the importance of this class of compounds, the synthesis of the title compound was undertaken and its crystal structure is reported herein.

## Structural commentary   

**Figure 1:**
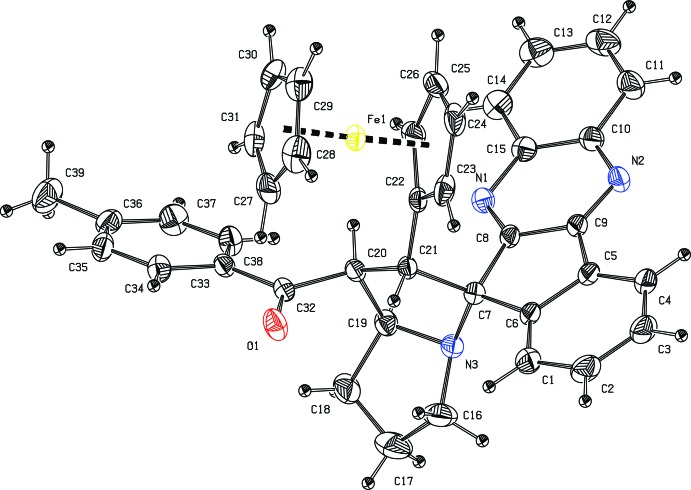
The mol­ecular structure of the title compound, with displacement ellipsoids drawn at the 30% probability level. H atoms are shown as small spheres of arbitrary radius.

In the title compound (Fig. 1 ▶), the four-fused-rings system of the 11*H*-indeno­[1,2-*b*]quinoxaline unit is approximately planar [maximum deviation = 0.167 (4) Å for C13] and forms a dihedral angle of 37.25 (6)° with the C33–C38 benzene ring of the methyl­benzoyl group. In the fused pyrrolidine system, both five-membered rings adopt a twist conformation, as indicated by the puckering parameters (Cremer & Pople, 1975 ▶) θ = 0.382 (3) Å, ϕ = 107.1 (4)° for C19/C18/C17/C16/N3 and θ = 0.359 (2) Å, ϕ = 106.1 (3)° for C19/C20/C21/C7/N3. The dihedral angle between the least-squares mean planes through the pyrrolidine rings is 56.89 (7)°. The mean plane through the C19/C20/C21/C7/N3 pyrrolidine ring is nearly orthogonal to the C5/C6/C7/C8/C9 cyclo­pentane ring, forming a dihedral angle of 88.84 (8)°. The dihedral angle between the cyclo­pentane rings in the ferrocene fragment is 2.18 (8)°. Bond lengths and angles are not unusual and in good agreement with those recently reported for the related compound 2-(4-bromo­benzo­yl)-1-ferrocenyl­spiro­[11*H*-pyrrolidizine-3,11′-indeno­[1,2-*b*]quinoxaline] (Suhitha *et al.*, 2013 ▶). The mol­ecular conformation is stabilized by an intra­molecular C—H⋯O hydrogen bond (Table 1 ▶).

## Supra­molecular features   

**Figure 2:**
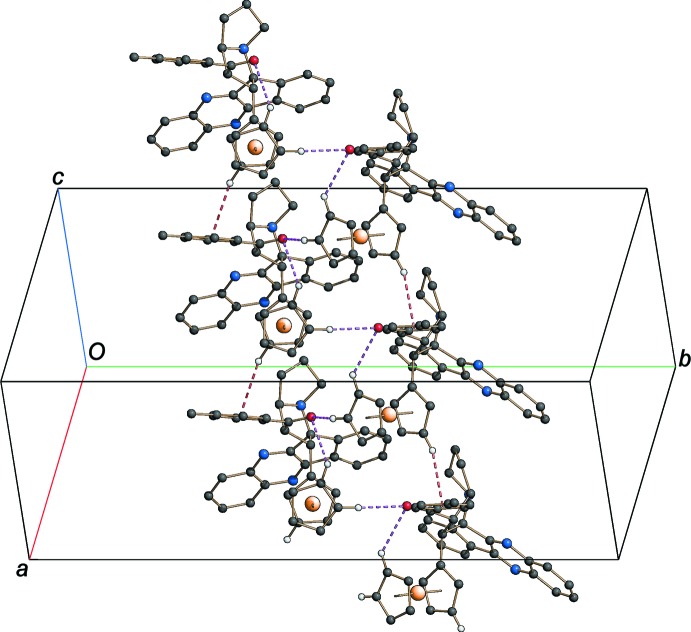
Partial crystal packing of the title compound, showing the formation of a double chain running parallel to the *c* axis *via* C—H⋯O hydrogen bonds (violet dashed lines) and C—H⋯π inter­actions (red dashed lines). H atoms not involved in hydrogen-bond inter­actions have been omitted.

In the crystal structure, mol­ecules are linked into double chains running parallel to the *c* axis by inter­molecular non-classical C—H⋯O hydrogen bonds and weak C—H⋯π inter­actions (Table 1 ▶) involving H atoms of the cyclo­penta­dienyl groups as donors (Fig. 2 ▶).

## Synthesis and crystallization   

Ninhydrin (1 mmol) and 1,2-phenyl­enedi­amine (1 mmol) were mixed and stirred with methanol (10 ml) for 10 min. To this mixture, proline (1 mmol) and 1-ferrocenyl-3-(4-methyl­benzo­yl)prop-2-ene dipolarophile (1 mmol) were added and refluxed up to the end of the reaction as observed by thin-layer chromatography. The solvent was removed from the mixture under reduced pressure and the crude product was obtained using column chromatography. The crude extract was purified by petroleum ether and ethyl acetate (4:1 *v*/*v*). Single crystals suitable for the X-ray diffraction analysis were obtained by slow evaporation of the solvent at room temperature.

## Refinement details   

Crystal data, data collection and structure refinement details are summarized in Table 2 ▶. All H atoms were placed in calculated positions, with C—H = 0.93–0.98 Å, and refined using a riding-model approximation, with *U*_iso_(H) = 1.5*U*_eq_(C) for methyl groups or 1.2*U*_eq_(C) otherwise. DELU restraints were applied to atoms C24 and C25.

## Supplementary Material

Crystal structure: contains datablock(s) I. DOI: 10.1107/S1600536814017644/rz5131sup1.cif

Structure factors: contains datablock(s) I. DOI: 10.1107/S1600536814017644/rz5131Isup2.hkl

CCDC reference: 1017369

Additional supporting information:  crystallographic information; 3D view; checkCIF report

## Figures and Tables

**Table 1:** Hydrogen-bond geometry (Å, °) *Cg*1 is the centroid of C33–C39 ring.

*D*—H⋯*A*	*D*—H	H⋯*A*	*D*⋯*A*	*D*—H⋯*A*
C27—H27⋯O1	0.98	2.57	3.332 (4)	134
C28—H28⋯O1^i^	0.98	2.55	3.474 (3)	157
C25—H25⋯*Cg*1^ii^	0.98	2.83	3.781 (3)	163

Symmetry codes: (i) 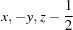; (ii) 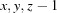.

**Table 2:** Experimental details

Crystal data	
Chemical formula	[Fe(C_5_H_5_)(C_34_H_28_N_3_O)]
*M*_r_	615.53
Crystal system, space group	Monoclinic, *C* *c*
Temperature (K)	293
*a*, *b*, *c* (Å)	12.0017 (4), 30.2487 (10), 9.3597 (3)
β (°)	116.179 (1)
*V* (Å^3^)	3049.35 (17)
*Z*	4
Radiation type	Mo *K*α
μ (mm^−1^)	0.53
Crystal size (mm)	0.35 × 0.30 × 0.25

Data collection	
Diffractometer	Bruker Kappa APEXII CCD
Absorption correction	Multi-scan (*SADABS*; Bruker, 2004 ▶)
*T*_min_, *T*_max_	0.836, 0.879
No. of measured, independent and observed [*I* > 2σ(*I*)] reflections	17682, 5362, 5128
*R*_int_	0.021
(sin θ/λ)_max_ (Å^−1^)	0.595

Refinement	
*R*[*F* ^2^ > 2σ(*F* ^2^)], *wR*(*F* ^2^), *S*	0.024, 0.062, 1.03
No. of reflections	5362
No. of parameters	399
No. of restraints	3
H-atom treatment	H-atom parameters constrained
Δρ_max_, Δρ_min_ (e Å^−3^)	0.16, −0.15
Absolute structure	Flack (1983 ▶), 2669 Friedel pairs
Absolute structure parameter	−0.007 (9)

Computer programs: *APEX2*, *SAINT* and *XPREP* (Bruker, 2004 ▶), *SHELXS97* and *SHELXL97* (Sheldrick, 2008 ▶), *ORTEP-3 for Windows* (Farrugia, 2012 ▶), *SCHAKAL99* (Keller, 1999 ▶) and *publCIF* (Westrip, 2010 ▶).

## supplementary crystallographic information

### Crystal data

**Table:**

[Fe(C_5_H_5_)(C_34_H_28_N_3_O)]	*Z* = 4
*M**_r_* = 615.53	*F*(000) = 1288
Monoclinic, *C**c*	*D*_x_ = 1.341 Mg m^−^^3^
Hall symbol: C -2yc	Mo *K*α radiation, λ = 0.71073 Å
*a* = 12.0017 (4) Å	θ = 4.8–56.4°
*b* = 30.2487 (10) Å	µ = 0.53 mm^−^^1^
*c* = 9.3597 (3) Å	*T* = 293 K
β = 116.179 (1)°	Block, colourless
*V* = 3049.35 (17) Å^3^	0.35 × 0.30 × 0.25 mm

### Data collection

**Table:**

Bruker Kappa APEXII CCD diffractometer	5362 independent reflections
Radiation source: fine-focus sealed tube	5128 reflections with *I* > 2σ(*I*)
Graphite monochromator	*R*_int_ = 0.021
Bruker axs kappa apex2 CCD Diffractometer scans	θ_max_ = 25.0°, θ_min_ = 2.4°
Absorption correction: multi-scan (*SADABS*; Bruker, 2004)	*h* = −14→14
*T*_min_ = 0.836, *T*_max_ = 0.879	*k* = −35→35
17682 measured reflections	*l* = −11→11

### Refinement

**Table:**

Refinement on *F*^2^	Hydrogen site location: inferred from neighbouring sites
Least-squares matrix: full	H-atom parameters constrained
*R*[*F*^2^ > 2σ(*F*^2^)] = 0.024	*w* = 1/[σ^2^(*F*_o_^2^) + (0.0347*P*)^2^ + 0.3631*P*] where *P* = (*F*_o_^2^ + 2*F*_c_^2^)/3
*wR*(*F*^2^) = 0.062	(Δ/σ)_max_ = 0.002
*S* = 1.03	Δρ_max_ = 0.16 e Å^−^^3^
5362 reflections	Δρ_min_ = −0.15 e Å^−^^3^
399 parameters	Extinction correction: *SHELXL97* (Sheldrick, 2008), Fc^*^=kFc[1+0.001xFc^2^λ^3^/sin(2θ)]^-1/4^
3 restraints	Extinction coefficient: 0.00070 (15)
Primary atom site location: structure-invariant direct methods	Absolute structure: Flack (1983), 2669 Friedel pairs
Secondary atom site location: difference Fourier map	Absolute structure parameter: −0.007 (9)

### Special details

**Table:**

Geometry. All e.s.d.'s (except the e.s.d. in the dihedral angle between two l.s. planes) are estimated using the full covariance matrix. The cell e.s.d.'s are taken into account individually in the estimation of e.s.d.'s in distances, angles and torsion angles; correlations between e.s.d.'s in cell parameters are only used when they are defined by crystal symmetry. An approximate (isotropic) treatment of cell e.s.d.'s is used for estimating e.s.d.'s involving l.s. planes.
Refinement. Refinement of *F*^2^ against ALL reflections. The weighted *R*-factor *wR* and goodness of fit *S* are based on *F*^2^, conventional *R*-factors *R* are based on *F*, with *F* set to zero for negative *F*^2^. The threshold expression of *F*^2^ > σ(*F*^2^) is used only for calculating *R*-factors(gt) *etc*. and is not relevant to the choice of reflections for refinement. *R*-factors based on *F*^2^ are statistically about twice as large as those based on *F*, and *R*- factors based on all data will be even larger.

### Fractional atomic coordinates and isotropic or equivalent isotropic displacement parameters (Å^2^)

**Table:**

	*x*	*y*	*z*	*U*_iso_*/*U*_eq_	
Fe1	0.01108 (2)	0.077233 (8)	0.28428 (2)	0.03569 (8)	
O1	−0.07741 (15)	0.06919 (5)	0.67565 (18)	0.0530 (4)	
N1	−0.30744 (14)	0.19423 (5)	0.21696 (19)	0.0373 (3)	
N2	−0.47392 (15)	0.17673 (6)	−0.10980 (19)	0.0427 (4)	
N3	−0.39342 (15)	0.13086 (5)	0.39825 (19)	0.0398 (4)	
C1	−0.47492 (19)	0.04151 (7)	0.1670 (3)	0.0483 (5)	
H1	−0.4398	0.0283	0.2669	0.058*	
C2	−0.5562 (2)	0.01800 (8)	0.0334 (3)	0.0567 (6)	
H2	−0.5768	−0.0110	0.0449	0.068*	
C3	−0.6066 (2)	0.03731 (8)	−0.1163 (3)	0.0567 (6)	
H3	−0.6608	0.0211	−0.2038	0.068*	
C4	−0.57798 (19)	0.07972 (7)	−0.1372 (3)	0.0469 (5)	
H4	−0.6109	0.0923	−0.2381	0.056*	
C5	−0.49823 (16)	0.10371 (7)	−0.0040 (2)	0.0370 (4)	
C6	−0.44732 (16)	0.08500 (6)	0.1482 (2)	0.0352 (4)	
C7	−0.35942 (16)	0.11702 (6)	0.2715 (2)	0.0332 (4)	
C8	−0.36774 (15)	0.15702 (6)	0.1682 (2)	0.0326 (4)	
C9	−0.44990 (16)	0.14829 (6)	0.0059 (2)	0.0351 (4)	
C10	−0.40833 (18)	0.21570 (7)	−0.0633 (2)	0.0419 (5)	
C11	−0.4220 (2)	0.24758 (8)	−0.1796 (3)	0.0579 (6)	
H11	−0.4772	0.2427	−0.2856	0.070*	
C12	−0.3537 (2)	0.28584 (8)	−0.1361 (3)	0.0646 (7)	
H12	−0.3623	0.3067	−0.2133	0.078*	
C13	−0.2712 (3)	0.29375 (8)	0.0231 (3)	0.0629 (6)	
H13	−0.2250	0.3197	0.0504	0.075*	
C14	−0.2576 (2)	0.26398 (7)	0.1388 (3)	0.0521 (5)	
H14	−0.2034	0.2699	0.2445	0.063*	
C15	−0.32574 (17)	0.22426 (6)	0.0982 (2)	0.0400 (4)	
C16	−0.4350 (3)	0.09612 (10)	0.4739 (3)	0.0665 (7)	
H16A	−0.3873	0.0692	0.4885	0.080*	
H16B	−0.5224	0.0896	0.4115	0.080*	
C17	−0.4115 (3)	0.11636 (12)	0.6293 (4)	0.0865 (10)	
H17A	−0.4050	0.0938	0.7062	0.104*	
H17B	−0.4776	0.1367	0.6173	0.104*	
C18	−0.2913 (2)	0.14035 (10)	0.6803 (3)	0.0665 (7)	
H18A	−0.2226	0.1221	0.7513	0.080*	
H18B	−0.2917	0.1676	0.7349	0.080*	
C19	−0.28009 (18)	0.15018 (7)	0.5261 (2)	0.0410 (4)	
H19	−0.2823	0.1823	0.5111	0.049*	
C20	−0.16588 (16)	0.13083 (6)	0.5049 (2)	0.0321 (4)	
H20	−0.1249	0.1552	0.4778	0.039*	
C21	−0.22308 (16)	0.09977 (6)	0.3613 (2)	0.0313 (4)	
H21	−0.2252	0.0698	0.4001	0.038*	
C22	−0.16107 (16)	0.09780 (7)	0.2525 (2)	0.0349 (4)	
C23	−0.17042 (19)	0.06193 (9)	0.1483 (3)	0.0512 (6)	
H23	−0.2096	0.0332	0.1445	0.061*	
C24	−0.1133 (2)	0.07513 (9)	0.0524 (3)	0.0606 (7)	
H24	−0.1058	0.0569	−0.0295	0.073*	
C25	−0.0684 (2)	0.11832 (9)	0.0928 (3)	0.0581 (6)	
H25	−0.0247	0.1355	0.0444	0.070*	
C26	−0.09819 (18)	0.13257 (7)	0.2151 (3)	0.0433 (5)	
H26	−0.0769	0.1614	0.2680	0.052*	
C27	0.0989 (2)	0.04430 (9)	0.4942 (3)	0.0587 (6)	
H27	0.0606	0.0336	0.5608	0.070*	
C28	0.1084 (2)	0.02060 (7)	0.3691 (3)	0.0622 (7)	
H28	0.0777	−0.0094	0.3345	0.075*	
C29	0.1692 (2)	0.04753 (7)	0.3035 (3)	0.0549 (6)	
H29	0.1885	0.0397	0.2153	0.066*	
C30	0.19731 (19)	0.08743 (8)	0.3866 (3)	0.0512 (6)	
H30	0.2394	0.1126	0.3657	0.061*	
C31	0.1536 (2)	0.08591 (9)	0.5048 (3)	0.0531 (6)	
H31	0.1608	0.1094	0.5804	0.064*	
C32	−0.07122 (18)	0.10845 (6)	0.6539 (2)	0.0347 (4)	
C33	0.02945 (18)	0.13584 (6)	0.7763 (2)	0.0351 (4)	
C34	0.1321 (2)	0.11481 (7)	0.8939 (2)	0.0437 (5)	
H34	0.1369	0.0841	0.8950	0.052*	
C35	0.22726 (19)	0.13901 (8)	1.0094 (3)	0.0486 (5)	
H35	0.2955	0.1244	1.0863	0.058*	
C36	0.2222 (2)	0.18463 (8)	1.0119 (3)	0.0515 (5)	
C37	0.1191 (2)	0.20554 (7)	0.8961 (3)	0.0575 (6)	
H37	0.1135	0.2362	0.8967	0.069*	
C38	0.0244 (2)	0.18150 (6)	0.7795 (3)	0.0479 (5)	
H38	−0.0435	0.1962	0.7024	0.057*	
C39	0.3290 (2)	0.21089 (11)	1.1340 (3)	0.0760 (8)	
H39A	0.3816	0.1916	1.2184	0.114*	
H39B	0.2971	0.2338	1.1767	0.114*	
H39C	0.3760	0.2239	1.0844	0.114*	

### Atomic displacement parameters (Å^2^)

**Table:**

	*U*^11^	*U*^22^	*U*^33^	*U*^12^	*U*^13^	*U*^23^
Fe1	0.03174 (13)	0.04076 (14)	0.02999 (13)	0.00726 (12)	0.00945 (10)	−0.00137 (13)
O1	0.0648 (10)	0.0386 (8)	0.0384 (8)	−0.0019 (7)	0.0072 (8)	0.0075 (6)
N1	0.0373 (8)	0.0346 (8)	0.0374 (8)	0.0060 (7)	0.0141 (7)	0.0034 (7)
N2	0.0392 (9)	0.0513 (9)	0.0333 (9)	0.0067 (7)	0.0120 (7)	0.0089 (7)
N3	0.0347 (8)	0.0528 (10)	0.0333 (8)	0.0024 (7)	0.0161 (7)	0.0008 (7)
C1	0.0442 (12)	0.0484 (11)	0.0473 (12)	−0.0071 (9)	0.0156 (10)	0.0039 (10)
C2	0.0516 (13)	0.0498 (13)	0.0662 (15)	−0.0198 (10)	0.0235 (12)	−0.0083 (11)
C3	0.0470 (13)	0.0692 (15)	0.0490 (14)	−0.0231 (11)	0.0167 (11)	−0.0176 (12)
C4	0.0345 (10)	0.0655 (14)	0.0357 (11)	−0.0089 (10)	0.0110 (9)	−0.0044 (10)
C5	0.0260 (9)	0.0473 (11)	0.0364 (10)	−0.0005 (8)	0.0127 (8)	−0.0006 (8)
C6	0.0242 (9)	0.0455 (11)	0.0333 (10)	−0.0003 (7)	0.0105 (8)	−0.0007 (8)
C7	0.0310 (9)	0.0354 (9)	0.0319 (9)	0.0003 (7)	0.0127 (8)	0.0009 (8)
C8	0.0249 (8)	0.0398 (10)	0.0308 (9)	0.0056 (7)	0.0102 (7)	0.0019 (8)
C9	0.0265 (9)	0.0458 (11)	0.0317 (9)	0.0036 (7)	0.0117 (8)	0.0029 (8)
C10	0.0388 (10)	0.0432 (11)	0.0455 (12)	0.0110 (9)	0.0202 (9)	0.0122 (9)
C11	0.0572 (14)	0.0612 (14)	0.0539 (14)	0.0146 (11)	0.0231 (12)	0.0213 (11)
C12	0.0754 (16)	0.0483 (13)	0.0813 (19)	0.0135 (12)	0.0446 (15)	0.0291 (13)
C13	0.0687 (16)	0.0388 (11)	0.0858 (19)	0.0051 (11)	0.0383 (15)	0.0140 (12)
C14	0.0532 (12)	0.0374 (11)	0.0643 (14)	0.0050 (9)	0.0246 (11)	0.0031 (10)
C15	0.0372 (10)	0.0383 (10)	0.0472 (12)	0.0099 (8)	0.0211 (9)	0.0050 (9)
C16	0.0757 (17)	0.0808 (17)	0.0611 (16)	−0.0188 (14)	0.0467 (15)	−0.0029 (13)
C17	0.098 (2)	0.120 (3)	0.0646 (19)	−0.0150 (19)	0.0563 (18)	0.0006 (17)
C18	0.0567 (14)	0.110 (2)	0.0376 (13)	0.0111 (14)	0.0251 (11)	−0.0015 (13)
C19	0.0408 (10)	0.0474 (11)	0.0337 (10)	0.0069 (9)	0.0155 (9)	−0.0003 (9)
C20	0.0350 (9)	0.0329 (9)	0.0258 (9)	−0.0009 (7)	0.0109 (8)	0.0020 (7)
C21	0.0291 (9)	0.0329 (9)	0.0274 (9)	0.0020 (7)	0.0082 (7)	0.0031 (7)
C22	0.0266 (9)	0.0447 (10)	0.0267 (10)	0.0073 (8)	0.0057 (8)	0.0036 (8)
C23	0.0336 (11)	0.0722 (15)	0.0353 (12)	0.0000 (10)	0.0039 (9)	−0.0194 (11)
C24	0.0422 (13)	0.1079 (17)	0.0259 (12)	0.0157 (11)	0.0098 (11)	−0.0101 (12)
C25	0.0455 (12)	0.0903 (14)	0.0424 (13)	0.0241 (11)	0.0230 (11)	0.0269 (11)
C26	0.0388 (11)	0.0508 (12)	0.0423 (11)	0.0167 (9)	0.0198 (9)	0.0170 (9)
C27	0.0497 (13)	0.0673 (15)	0.0489 (14)	0.0198 (12)	0.0124 (11)	0.0250 (12)
C28	0.0623 (15)	0.0347 (11)	0.0722 (17)	0.0160 (11)	0.0137 (13)	0.0040 (11)
C29	0.0465 (12)	0.0565 (13)	0.0574 (14)	0.0210 (10)	0.0191 (11)	−0.0052 (11)
C30	0.0300 (10)	0.0560 (13)	0.0595 (16)	0.0058 (9)	0.0123 (11)	−0.0027 (12)
C31	0.0386 (12)	0.0655 (15)	0.0373 (13)	0.0129 (11)	0.0004 (10)	−0.0112 (11)
C32	0.0371 (10)	0.0379 (10)	0.0269 (9)	0.0031 (8)	0.0122 (8)	0.0030 (8)
C33	0.0353 (10)	0.0424 (9)	0.0269 (9)	0.0028 (8)	0.0131 (8)	0.0017 (8)
C34	0.0417 (11)	0.0486 (12)	0.0368 (11)	0.0050 (9)	0.0136 (9)	−0.0025 (9)
C35	0.0315 (10)	0.0676 (14)	0.0395 (12)	0.0051 (10)	0.0090 (9)	−0.0035 (10)
C36	0.0408 (11)	0.0716 (15)	0.0437 (12)	−0.0157 (10)	0.0201 (10)	−0.0145 (11)
C37	0.0654 (15)	0.0433 (12)	0.0574 (14)	−0.0087 (10)	0.0213 (13)	−0.0075 (11)
C38	0.0483 (12)	0.0443 (10)	0.0404 (10)	0.0028 (11)	0.0098 (9)	0.0030 (11)
C39	0.0551 (15)	0.0917 (19)	0.0712 (17)	−0.0232 (14)	0.0187 (13)	−0.0278 (15)

### Geometric parameters (Å, º)

**Table:**

Fe1—C24	2.021 (2)	C17—H17A	0.9700
Fe1—C28	2.028 (2)	C17—H17B	0.9700
Fe1—C30	2.030 (2)	C18—C19	1.536 (3)
Fe1—C23	2.033 (2)	C18—H18A	0.9700
Fe1—C27	2.034 (2)	C18—H18B	0.9700
Fe1—C31	2.035 (2)	C19—C20	1.582 (3)
Fe1—C29	2.035 (2)	C19—H19	0.9800
Fe1—C25	2.039 (2)	C20—C32	1.515 (2)
Fe1—C26	2.0473 (19)	C20—C21	1.532 (2)
Fe1—C22	2.0500 (18)	C20—H20	0.9800
O1—C32	1.213 (2)	C21—C22	1.504 (3)
N1—C8	1.307 (2)	C21—H21	0.9800
N1—C15	1.376 (2)	C22—C26	1.425 (3)
N2—C9	1.312 (2)	C22—C23	1.430 (3)
N2—C10	1.377 (3)	C23—C24	1.407 (4)
N3—C16	1.472 (3)	C23—H23	0.9800
N3—C7	1.475 (2)	C24—C25	1.400 (4)
N3—C19	1.479 (3)	C24—H24	0.9800
C1—C6	1.386 (3)	C25—C26	1.409 (3)
C1—C2	1.394 (3)	C25—H25	0.9800
C1—H1	0.9300	C26—H26	0.9800
C2—C3	1.387 (3)	C27—C31	1.402 (4)
C2—H2	0.9300	C27—C28	1.419 (4)
C3—C4	1.364 (3)	C27—H27	0.9800
C3—H3	0.9300	C28—C29	1.403 (4)
C4—C5	1.394 (3)	C28—H28	0.9800
C4—H4	0.9300	C29—C30	1.394 (3)
C5—C6	1.399 (3)	C29—H29	0.9800
C5—C9	1.455 (3)	C30—C31	1.420 (4)
C6—C7	1.519 (3)	C30—H30	0.9800
C7—C8	1.524 (3)	C31—H31	0.9800
C7—C21	1.563 (2)	C32—C33	1.496 (3)
C8—C9	1.424 (3)	C33—C38	1.384 (3)
C10—C11	1.409 (3)	C33—C34	1.392 (3)
C10—C15	1.418 (3)	C34—C35	1.385 (3)
C11—C12	1.372 (3)	C34—H34	0.9300
C11—H11	0.9300	C35—C36	1.382 (3)
C12—C13	1.399 (4)	C35—H35	0.9300
C12—H12	0.9300	C36—C37	1.387 (3)
C13—C14	1.361 (3)	C36—C39	1.512 (3)
C13—H13	0.9300	C37—C38	1.385 (3)
C14—C15	1.408 (3)	C37—H37	0.9300
C14—H14	0.9300	C38—H38	0.9300
C16—C17	1.485 (4)	C39—H39A	0.9600
C16—H16A	0.9700	C39—H39B	0.9600
C16—H16B	0.9700	C39—H39C	0.9600
C17—C18	1.492 (4)		
			
C24—Fe1—C28	114.77 (11)	C17—C18—H18A	110.7
C24—Fe1—C30	130.48 (11)	C19—C18—H18A	110.7
C28—Fe1—C30	67.62 (10)	C17—C18—H18B	110.7
C24—Fe1—C23	40.62 (11)	C19—C18—H18B	110.7
C28—Fe1—C23	109.02 (10)	H18A—C18—H18B	108.8
C30—Fe1—C23	169.36 (10)	N3—C19—C18	104.69 (18)
C24—Fe1—C27	147.63 (12)	N3—C19—C20	106.90 (14)
C28—Fe1—C27	40.90 (11)	C18—C19—C20	119.08 (17)
C30—Fe1—C27	67.97 (11)	N3—C19—H19	108.6
C23—Fe1—C27	116.67 (11)	C18—C19—H19	108.6
C24—Fe1—C31	170.27 (13)	C20—C19—H19	108.6
C28—Fe1—C31	68.32 (10)	C32—C20—C21	113.42 (14)
C30—Fe1—C31	40.89 (11)	C32—C20—C19	112.91 (15)
C23—Fe1—C31	148.57 (11)	C21—C20—C19	105.04 (14)
C27—Fe1—C31	40.32 (10)	C32—C20—H20	108.4
C24—Fe1—C29	107.41 (10)	C21—C20—H20	108.4
C28—Fe1—C29	40.40 (10)	C19—C20—H20	108.4
C30—Fe1—C29	40.12 (9)	C22—C21—C20	117.01 (15)
C23—Fe1—C29	130.92 (9)	C22—C21—C7	111.26 (14)
C27—Fe1—C29	68.42 (10)	C20—C21—C7	102.99 (13)
C31—Fe1—C29	68.50 (10)	C22—C21—H21	108.4
C24—Fe1—C25	40.34 (10)	C20—C21—H21	108.4
C28—Fe1—C25	145.53 (11)	C7—C21—H21	108.4
C30—Fe1—C25	108.23 (10)	C26—C22—C23	106.43 (19)
C23—Fe1—C25	68.41 (11)	C26—C22—C21	127.66 (18)
C27—Fe1—C25	171.78 (12)	C23—C22—C21	125.33 (19)
C31—Fe1—C25	132.14 (12)	C26—C22—Fe1	69.54 (11)
C29—Fe1—C25	113.78 (10)	C23—C22—Fe1	68.86 (11)
C24—Fe1—C26	67.63 (10)	C21—C22—Fe1	132.99 (12)
C28—Fe1—C26	173.02 (11)	C24—C23—C22	107.8 (2)
C30—Fe1—C26	116.33 (9)	C24—C23—Fe1	69.23 (14)
C23—Fe1—C26	68.17 (10)	C22—C23—Fe1	70.14 (11)
C27—Fe1—C26	133.86 (9)	C24—C23—H23	126.1
C31—Fe1—C26	110.48 (9)	C22—C23—H23	126.1
C29—Fe1—C26	146.24 (9)	Fe1—C23—H23	126.1
C25—Fe1—C26	40.33 (9)	C25—C24—C23	109.3 (2)
C24—Fe1—C22	68.55 (9)	C25—C24—Fe1	70.53 (14)
C28—Fe1—C22	133.07 (10)	C23—C24—Fe1	70.15 (13)
C30—Fe1—C22	148.43 (8)	C25—C24—H24	125.4
C23—Fe1—C22	40.99 (8)	C23—C24—H24	125.4
C27—Fe1—C22	110.41 (9)	Fe1—C24—H24	125.4
C31—Fe1—C22	116.75 (9)	C24—C25—C26	107.4 (2)
C29—Fe1—C22	171.09 (9)	C24—C25—Fe1	69.13 (15)
C25—Fe1—C22	68.71 (8)	C26—C25—Fe1	70.14 (12)
C26—Fe1—C22	40.71 (8)	C24—C25—H25	126.3
C8—N1—C15	114.85 (16)	C26—C25—H25	126.3
C9—N2—C10	114.42 (16)	Fe1—C25—H25	126.3
C16—N3—C7	117.20 (17)	C25—C26—C22	109.0 (2)
C16—N3—C19	106.19 (17)	C25—C26—Fe1	69.52 (12)
C7—N3—C19	106.32 (14)	C22—C26—Fe1	69.74 (11)
C6—C1—C2	118.8 (2)	C25—C26—H26	125.5
C6—C1—H1	120.6	C22—C26—H26	125.5
C2—C1—H1	120.6	Fe1—C26—H26	125.5
C3—C2—C1	120.8 (2)	C31—C27—C28	107.9 (2)
C3—C2—H2	119.6	C31—C27—Fe1	69.88 (13)
C1—C2—H2	119.6	C28—C27—Fe1	69.33 (13)
C4—C3—C2	121.0 (2)	C31—C27—H27	126.1
C4—C3—H3	119.5	C28—C27—H27	126.1
C2—C3—H3	119.5	Fe1—C27—H27	126.1
C3—C4—C5	118.6 (2)	C29—C28—C27	108.3 (2)
C3—C4—H4	120.7	C29—C28—Fe1	70.07 (12)
C5—C4—H4	120.7	C27—C28—Fe1	69.76 (12)
C4—C5—C6	121.28 (19)	C29—C28—H28	125.9
C4—C5—C9	129.57 (18)	C27—C28—H28	125.9
C6—C5—C9	109.10 (16)	Fe1—C28—H28	125.9
C1—C6—C5	119.44 (18)	C30—C29—C28	107.6 (2)
C1—C6—C7	129.30 (18)	C30—C29—Fe1	69.74 (12)
C5—C6—C7	111.14 (16)	C28—C29—Fe1	69.53 (13)
N3—C7—C6	116.47 (15)	C30—C29—H29	126.2
N3—C7—C8	108.71 (14)	C28—C29—H29	126.2
C6—C7—C8	101.26 (14)	Fe1—C29—H29	126.2
N3—C7—C21	104.93 (14)	C29—C30—C31	109.0 (2)
C6—C7—C21	114.01 (14)	C29—C30—Fe1	70.14 (13)
C8—C7—C21	111.49 (14)	C31—C30—Fe1	69.74 (13)
N1—C8—C9	123.25 (17)	C29—C30—H30	125.5
N1—C8—C7	126.25 (16)	C31—C30—H30	125.5
C9—C8—C7	110.48 (15)	Fe1—C30—H30	125.5
N2—C9—C8	123.75 (17)	C27—C31—C30	107.2 (2)
N2—C9—C5	128.23 (18)	C27—C31—Fe1	69.80 (13)
C8—C9—C5	108.02 (16)	C30—C31—Fe1	69.37 (13)
N2—C10—C11	119.08 (19)	C27—C31—H31	126.4
N2—C10—C15	121.78 (17)	C30—C31—H31	126.4
C11—C10—C15	119.1 (2)	Fe1—C31—H31	126.4
C12—C11—C10	119.8 (2)	O1—C32—C33	120.16 (17)
C12—C11—H11	120.1	O1—C32—C20	121.03 (18)
C10—C11—H11	120.1	C33—C32—C20	118.80 (16)
C11—C12—C13	120.7 (2)	C38—C33—C34	118.1 (2)
C11—C12—H12	119.7	C38—C33—C32	122.76 (19)
C13—C12—H12	119.7	C34—C33—C32	119.13 (16)
C14—C13—C12	120.9 (2)	C35—C34—C33	120.86 (19)
C14—C13—H13	119.5	C35—C34—H34	119.6
C12—C13—H13	119.5	C33—C34—H34	119.6
C13—C14—C15	119.9 (2)	C36—C35—C34	120.9 (2)
C13—C14—H14	120.1	C36—C35—H35	119.5
C15—C14—H14	120.1	C34—C35—H35	119.5
N1—C15—C14	118.58 (19)	C35—C36—C37	118.2 (2)
N1—C15—C10	121.85 (17)	C35—C36—C39	120.6 (2)
C14—C15—C10	119.55 (19)	C37—C36—C39	121.2 (2)
N3—C16—C17	102.6 (2)	C38—C37—C36	121.0 (2)
N3—C16—H16A	111.2	C38—C37—H37	119.5
C17—C16—H16A	111.2	C36—C37—H37	119.5
N3—C16—H16B	111.2	C33—C38—C37	120.8 (2)
C17—C16—H16B	111.2	C33—C38—H38	119.6
H16A—C16—H16B	109.2	C37—C38—H38	119.6
C16—C17—C18	104.7 (2)	C36—C39—H39A	109.5
C16—C17—H17A	110.8	C36—C39—H39B	109.5
C18—C17—H17A	110.8	H39A—C39—H39B	109.5
C16—C17—H17B	110.8	C36—C39—H39C	109.5
C18—C17—H17B	110.8	H39A—C39—H39C	109.5
H17A—C17—H17B	108.9	H39B—C39—H39C	109.5
C17—C18—C19	105.4 (2)		
			
C6—C1—C2—C3	1.4 (3)	C22—Fe1—C24—C23	−38.04 (14)
C1—C2—C3—C4	0.2 (4)	C23—C24—C25—C26	−0.3 (3)
C2—C3—C4—C5	−1.1 (3)	Fe1—C24—C25—C26	−59.95 (15)
C3—C4—C5—C6	0.5 (3)	C23—C24—C25—Fe1	59.65 (17)
C3—C4—C5—C9	177.6 (2)	C28—Fe1—C25—C24	55.1 (2)
C2—C1—C6—C5	−2.0 (3)	C30—Fe1—C25—C24	131.93 (15)
C2—C1—C6—C7	−177.6 (2)	C23—Fe1—C25—C24	−37.33 (15)
C4—C5—C6—C1	1.1 (3)	C31—Fe1—C25—C24	171.09 (17)
C9—C5—C6—C1	−176.54 (17)	C29—Fe1—C25—C24	89.21 (16)
C4—C5—C6—C7	177.49 (17)	C26—Fe1—C25—C24	−118.6 (2)
C9—C5—C6—C7	−0.2 (2)	C22—Fe1—C25—C24	−81.52 (15)
C16—N3—C7—C6	45.0 (2)	C24—Fe1—C25—C26	118.6 (2)
C19—N3—C7—C6	163.50 (15)	C28—Fe1—C25—C26	173.73 (17)
C16—N3—C7—C8	158.52 (18)	C30—Fe1—C25—C26	−109.48 (14)
C19—N3—C7—C8	−82.99 (17)	C23—Fe1—C25—C26	81.26 (15)
C16—N3—C7—C21	−82.1 (2)	C31—Fe1—C25—C26	−70.32 (18)
C19—N3—C7—C21	36.39 (18)	C29—Fe1—C25—C26	−152.20 (14)
C1—C6—C7—N3	−66.0 (3)	C22—Fe1—C25—C26	37.07 (13)
C5—C6—C7—N3	118.03 (17)	C24—C25—C26—C22	0.6 (2)
C1—C6—C7—C8	176.3 (2)	Fe1—C25—C26—C22	−58.69 (13)
C5—C6—C7—C8	0.35 (19)	C24—C25—C26—Fe1	59.31 (16)
C1—C6—C7—C21	56.4 (3)	C23—C22—C26—C25	−0.7 (2)
C5—C6—C7—C21	−119.49 (17)	C21—C22—C26—C25	−172.28 (17)
C15—N1—C8—C9	−2.2 (2)	Fe1—C22—C26—C25	58.56 (14)
C15—N1—C8—C7	176.29 (16)	C23—C22—C26—Fe1	−59.25 (13)
N3—C7—C8—N1	57.8 (2)	C21—C22—C26—Fe1	129.16 (18)
C6—C7—C8—N1	−179.06 (17)	C24—Fe1—C26—C25	−37.92 (15)
C21—C7—C8—N1	−57.4 (2)	C30—Fe1—C26—C25	87.50 (17)
N3—C7—C8—C9	−123.62 (16)	C23—Fe1—C26—C25	−81.92 (16)
C6—C7—C8—C9	−0.43 (18)	C27—Fe1—C26—C25	171.48 (17)
C21—C7—C8—C9	121.19 (15)	C31—Fe1—C26—C25	131.82 (17)
C10—N2—C9—C8	2.9 (3)	C29—Fe1—C26—C25	50.2 (2)
C10—N2—C9—C5	−176.79 (17)	C22—Fe1—C26—C25	−120.6 (2)
N1—C8—C9—N2	−0.7 (3)	C24—Fe1—C26—C22	82.64 (14)
C7—C8—C9—N2	−179.35 (16)	C30—Fe1—C26—C22	−151.93 (12)
N1—C8—C9—C5	179.05 (16)	C23—Fe1—C26—C22	38.65 (13)
C7—C8—C9—C5	0.37 (19)	C27—Fe1—C26—C22	−67.95 (17)
C4—C5—C9—N2	2.2 (3)	C31—Fe1—C26—C22	−107.62 (14)
C6—C5—C9—N2	179.57 (18)	C29—Fe1—C26—C22	170.73 (16)
C4—C5—C9—C8	−177.53 (19)	C25—Fe1—C26—C22	120.6 (2)
C6—C5—C9—C8	−0.1 (2)	C24—Fe1—C27—C31	170.5 (2)
C9—N2—C10—C11	176.55 (18)	C28—Fe1—C27—C31	119.2 (2)
C9—N2—C10—C15	−2.3 (3)	C30—Fe1—C27—C31	38.42 (14)
N2—C10—C11—C12	−177.5 (2)	C23—Fe1—C27—C31	−152.10 (14)
C15—C10—C11—C12	1.4 (3)	C29—Fe1—C27—C31	81.78 (16)
C10—C11—C12—C13	−0.6 (4)	C26—Fe1—C27—C31	−67.52 (19)
C11—C12—C13—C14	−0.6 (4)	C22—Fe1—C27—C31	−107.69 (15)
C12—C13—C14—C15	1.0 (4)	C24—Fe1—C27—C28	51.3 (3)
C8—N1—C15—C14	−175.64 (17)	C30—Fe1—C27—C28	−80.78 (16)
C8—N1—C15—C10	2.7 (2)	C23—Fe1—C27—C28	88.70 (17)
C13—C14—C15—N1	178.2 (2)	C31—Fe1—C27—C28	−119.2 (2)
C13—C14—C15—C10	−0.2 (3)	C29—Fe1—C27—C28	−37.43 (15)
N2—C10—C15—N1	−0.5 (3)	C26—Fe1—C27—C28	173.28 (15)
C11—C10—C15—N1	−179.31 (18)	C22—Fe1—C27—C28	133.11 (15)
N2—C10—C15—C14	177.81 (18)	C31—C27—C28—C29	0.2 (3)
C11—C10—C15—C14	−1.0 (3)	Fe1—C27—C28—C29	59.69 (16)
C7—N3—C16—C17	157.8 (2)	C31—C27—C28—Fe1	−59.46 (15)
C19—N3—C16—C17	39.2 (3)	C24—Fe1—C28—C29	88.07 (16)
N3—C16—C17—C18	−39.0 (3)	C30—Fe1—C28—C29	−37.61 (15)
C16—C17—C18—C19	24.4 (3)	C23—Fe1—C28—C29	131.59 (14)
C16—N3—C19—C18	−23.9 (2)	C27—Fe1—C28—C29	−119.3 (2)
C7—N3—C19—C18	−149.40 (17)	C31—Fe1—C28—C29	−81.88 (16)
C16—N3—C19—C20	103.32 (19)	C25—Fe1—C28—C29	52.3 (2)
C7—N3—C19—C20	−22.20 (19)	C22—Fe1—C28—C29	171.18 (14)
C17—C18—C19—N3	−0.5 (3)	C24—Fe1—C28—C27	−152.62 (16)
C17—C18—C19—C20	−119.9 (2)	C30—Fe1—C28—C27	81.71 (16)
N3—C19—C20—C32	−124.84 (16)	C23—Fe1—C28—C27	−109.10 (16)
C18—C19—C20—C32	−6.7 (3)	C31—Fe1—C28—C27	37.44 (15)
N3—C19—C20—C21	−0.76 (19)	C29—Fe1—C28—C27	119.3 (2)
C18—C19—C20—C21	117.4 (2)	C25—Fe1—C28—C27	171.58 (19)
C32—C20—C21—C22	−92.15 (19)	C22—Fe1—C28—C27	−69.50 (19)
C19—C20—C21—C22	144.09 (16)	C27—C28—C29—C30	0.1 (3)
C32—C20—C21—C7	145.49 (15)	Fe1—C28—C29—C30	59.56 (15)
C19—C20—C21—C7	21.73 (17)	C27—C28—C29—Fe1	−59.51 (16)
N3—C7—C21—C22	−162.14 (15)	C24—Fe1—C29—C30	133.14 (17)
C6—C7—C21—C22	69.26 (19)	C28—Fe1—C29—C30	−118.9 (2)
C8—C7—C21—C22	−44.6 (2)	C23—Fe1—C29—C30	171.79 (18)
N3—C7—C21—C20	−36.00 (17)	C27—Fe1—C29—C30	−80.99 (17)
C6—C7—C21—C20	−164.60 (15)	C31—Fe1—C29—C30	−37.49 (17)
C8—C7—C21—C20	81.50 (17)	C25—Fe1—C29—C30	90.42 (18)
C20—C21—C22—C26	−32.5 (2)	C26—Fe1—C29—C30	57.5 (2)
C7—C21—C22—C26	85.5 (2)	C24—Fe1—C29—C28	−108.00 (16)
C20—C21—C22—C23	157.43 (18)	C30—Fe1—C29—C28	118.9 (2)
C7—C21—C22—C23	−84.6 (2)	C23—Fe1—C29—C28	−69.34 (19)
C20—C21—C22—Fe1	64.2 (2)	C27—Fe1—C29—C28	37.88 (15)
C7—C21—C22—Fe1	−177.84 (14)	C31—Fe1—C29—C28	81.38 (16)
C24—Fe1—C22—C26	−80.20 (15)	C25—Fe1—C29—C28	−150.71 (15)
C28—Fe1—C22—C26	175.39 (15)	C26—Fe1—C29—C28	176.39 (17)
C30—Fe1—C22—C26	53.7 (2)	C28—C29—C30—C31	−0.3 (3)
C23—Fe1—C22—C26	−117.90 (19)	Fe1—C29—C30—C31	59.11 (16)
C27—Fe1—C22—C26	134.51 (13)	C28—C29—C30—Fe1	−59.43 (16)
C31—Fe1—C22—C26	90.85 (15)	C24—Fe1—C30—C29	−66.3 (2)
C25—Fe1—C22—C26	−36.74 (14)	C28—Fe1—C30—C29	37.87 (16)
C24—Fe1—C22—C23	37.71 (16)	C23—Fe1—C30—C29	−35.7 (7)
C28—Fe1—C22—C23	−66.71 (19)	C27—Fe1—C30—C29	82.21 (17)
C30—Fe1—C22—C23	171.6 (2)	C31—Fe1—C30—C29	120.1 (2)
C27—Fe1—C22—C23	−107.58 (16)	C25—Fe1—C30—C29	−105.55 (17)
C31—Fe1—C22—C23	−151.24 (16)	C22—Fe1—C30—C29	175.65 (16)
C25—Fe1—C22—C23	81.17 (16)	C24—Fe1—C30—C31	173.62 (16)
C26—Fe1—C22—C23	117.90 (19)	C28—Fe1—C30—C31	−82.25 (17)
C24—Fe1—C22—C21	156.9 (2)	C23—Fe1—C30—C31	−155.8 (6)
C28—Fe1—C22—C21	52.4 (2)	C27—Fe1—C30—C31	−37.90 (15)
C30—Fe1—C22—C21	−69.3 (3)	C29—Fe1—C30—C31	−120.1 (2)
C23—Fe1—C22—C21	119.2 (3)	C25—Fe1—C30—C31	134.33 (16)
C27—Fe1—C22—C21	11.6 (2)	C26—Fe1—C30—C31	91.43 (16)
C31—Fe1—C22—C21	−32.1 (2)	C22—Fe1—C30—C31	55.5 (2)
C25—Fe1—C22—C21	−159.7 (2)	C28—C27—C31—C30	−0.4 (2)
C26—Fe1—C22—C21	−122.9 (2)	Fe1—C27—C31—C30	−59.54 (16)
C26—C22—C23—C24	0.5 (2)	C28—C27—C31—Fe1	59.12 (16)
C21—C22—C23—C24	172.34 (18)	C29—C30—C31—C27	0.5 (3)
Fe1—C22—C23—C24	−59.19 (16)	Fe1—C30—C31—C27	59.82 (16)
C26—C22—C23—Fe1	59.69 (13)	C29—C30—C31—Fe1	−59.35 (16)
C21—C22—C23—Fe1	−128.47 (17)	C28—Fe1—C31—C27	−37.96 (15)
C28—Fe1—C23—C24	−106.18 (17)	C30—Fe1—C31—C27	−118.4 (2)
C30—Fe1—C23—C24	−36.4 (7)	C23—Fe1—C31—C27	53.3 (2)
C27—Fe1—C23—C24	−150.01 (16)	C29—Fe1—C31—C27	−81.56 (15)
C31—Fe1—C23—C24	174.5 (2)	C25—Fe1—C31—C27	175.25 (15)
C29—Fe1—C23—C24	−66.3 (2)	C26—Fe1—C31—C27	134.67 (14)
C25—Fe1—C23—C24	37.08 (15)	C22—Fe1—C31—C27	90.55 (16)
C26—Fe1—C23—C24	80.64 (16)	C28—Fe1—C31—C30	80.40 (16)
C22—Fe1—C23—C24	119.0 (2)	C23—Fe1—C31—C30	171.7 (2)
C24—Fe1—C23—C22	−119.0 (2)	C27—Fe1—C31—C30	118.4 (2)
C28—Fe1—C23—C22	134.79 (14)	C29—Fe1—C31—C30	36.80 (15)
C30—Fe1—C23—C22	−155.4 (6)	C25—Fe1—C31—C30	−66.39 (18)
C27—Fe1—C23—C22	90.96 (16)	C26—Fe1—C31—C30	−106.97 (14)
C31—Fe1—C23—C22	55.5 (3)	C22—Fe1—C31—C30	−151.09 (13)
C29—Fe1—C23—C22	174.69 (14)	C21—C20—C32—O1	−27.9 (3)
C25—Fe1—C23—C22	−81.95 (15)	C19—C20—C32—O1	91.5 (2)
C26—Fe1—C23—C22	−38.39 (13)	C21—C20—C32—C33	153.27 (16)
C22—C23—C24—C25	−0.1 (3)	C19—C20—C32—C33	−87.4 (2)
Fe1—C23—C24—C25	−59.89 (17)	O1—C32—C33—C38	−163.1 (2)
C22—C23—C24—Fe1	59.76 (14)	C20—C32—C33—C38	15.8 (3)
C28—Fe1—C24—C25	−149.24 (15)	O1—C32—C33—C34	15.9 (3)
C30—Fe1—C24—C25	−68.29 (18)	C20—C32—C33—C34	−165.23 (18)
C23—Fe1—C24—C25	120.0 (2)	C38—C33—C34—C35	−0.9 (3)
C27—Fe1—C24—C25	176.53 (18)	C32—C33—C34—C35	180.0 (2)
C29—Fe1—C24—C25	−106.48 (15)	C33—C34—C35—C36	0.7 (3)
C26—Fe1—C24—C25	37.92 (13)	C34—C35—C36—C37	0.2 (3)
C22—Fe1—C24—C25	81.95 (14)	C34—C35—C36—C39	−177.3 (2)
C28—Fe1—C24—C23	90.77 (17)	C35—C36—C37—C38	−0.9 (4)
C30—Fe1—C24—C23	171.71 (16)	C39—C36—C37—C38	176.7 (2)
C27—Fe1—C24—C23	56.5 (2)	C34—C33—C38—C37	0.3 (3)
C29—Fe1—C24—C23	133.53 (15)	C32—C33—C38—C37	179.3 (2)
C25—Fe1—C24—C23	−120.0 (2)	C36—C37—C38—C33	0.6 (4)
C26—Fe1—C24—C23	−82.07 (15)		

### Hydrogen-bond geometry (Å, º)

*Cg*1 is the centroid of C33–C39 ring.

**Table:**

*D*—H···*A*	*D*—H	H···*A*	*D*···*A*	*D*—H···*A*
C27—H27···O1	0.98	2.57	3.332 (4)	134
C28—H28···O1^i^	0.98	2.55	3.474 (3)	157
C25—H25···*Cg*1^ii^	0.98	2.83	3.781 (3)	163

Symmetry codes: (i) *x*, −*y*, *z*−1/2; (ii) *x*, *y*, *z*−1.
